# Predication of Writing Originality Based on Computational Linguistics

**DOI:** 10.3390/jintelligence10040124

**Published:** 2022-12-13

**Authors:** Liping Yang, Tao Xin, Sheng Zhang, Yunye Yu

**Affiliations:** 1 Collaborative Innovation Center of Assessment for Basic Education Quality, Beijing Normal University, Beijing 100091, China; 2 School of Foreign Languages, Southeast University, Nanjing 211189, China

**Keywords:** originality, assessing writing, automated scoring, topic analysis, semantic networks

## Abstract

Existing assessment methods of writing originality have been criticized for depending heavily on subjective scoring methods. This study attempted to investigate the use of topic analysis and semantic networks in assessing writing originality. Written material was collected from a Chinese language test administered to eighth-grade students. Two steps were performed: 1. Latent topics of essays in each writing task were identified, and essays on the same topic were treated as a refined reference group, within which an essay was to be evaluated; 2. A group of features was developed, including four categories, i.e., path distance, semantic differences, centrality, and similarity of the network drawn from each text response, which were used to quantify the differences among essays. The results show that writing originality scoring is not only related to the intrinsic characteristics of the text, but is also affected by the reference group in which it is to be evaluated. This study proves that computational linguistic features can be a predictor of originality in Chinese writing. Each feature type of the four categories can predict originality, although the effect varies across various topics. Furthermore, the feature analysis provided evidence and insights to human raters for originality scoring.

## 1. Introduction

Originality is an important construct in written creativity assessment. Scoring originality is difficult, and existing scoring methods have shifted from subjective assessment to objective assessment. One of the earliest methods is the Consensual Assessment Technique (CAT) (Amabile 1983; Cseh and Jeffries 2019; Kaufman et al. 2008), in which experienced writers or essay-scoring experts subjectively evaluate essays based on their own understanding of the topic and criteria for writing tasks. Compared to CAT, the rule-based scoring method makes the evaluation more transparent and objective (Mozaffari 2013). Rule-based scoring means that human raters evaluate creativity using a rubric, which usually consists of ordinal or interval scales to score different aspects of creative works. Rule-based scoring was used in the Torrance Test of Creative Thinking (TTCT, Torrance 1962) to assess students’ creativity. TTCT is different from Guilford’s tests of divergent production (Torrance 1966), and covers three aspects of English language writing: Fluency, flexibility, and originality, which is a common component of definitions of creativity. There are some divergent thinking tasks (e.g., writing beginnings and endings of a very short story are only scored for fluency (Barbot et al. 2016). In practice, descriptive statistics characterize the general features of the writings for human beings to rate the creativity level in student writings.

However, both of these human-scoring methods are labor-intensive, which limits their application, especially in large-scale writing assessments. The recent development of automated essay scoring systems (AESs) provides a solution to tackle the flaws inherent in human scoring, while new natural language processing technologies provide additional opportunities for analyzing the computational linguistic characteristics of writing originality.

Runco and Jaeger (2012) stated that the standard definition of creativity, which includes two criteria; namely, originality and effectiveness, is insufficient. Further work is needed especially in assessing originality, which is a crucial aspect of creativity. In the current study, we refer to Zedelius et al.’s (2019) definition of the relationship between originality and creativity, in which originality is seen as one of the three components (image, voice, and originality) of writing creativity. We define originality as “uncommonness”, which is a classical indicator of originality (Wilson et al. 1953; Zedelius et al. 2019). This definition of originality scoring focuses on assessing the uncommonness of the relative frequency of occurrence of plot lines of essay. We review the related studies below.

### 1.1. Automated Scoring of Creativity/Originality in Writing

With the development of text analysis technology, systems for automated essay scoring have been widely used in writing evaluation (Burstein et al. 2013; Page 1994; Schultz 2013; Mayfield and Rosé 2013; Foltz et al. 2013; Rudner and Liang 2002). In general, AES can be used to provide objective evidence derived from the essay to supplement human scoring in large-scale examinations or to replace human scoring in daily writing practice. Contemporary state-of-the-art AES systems can achieve stellar performance in predicting certain aspects of writing, including vocabulary, grammar, language accuracy, and discourse structure, with correlations of approximately 0.70–0.80 between automated scores and human-ratings. However, there is room for improvement in terms of analyzing the latent traits of essays, such as content.

Compared to advances in AES, the automated scoring of writing creativity lags behind. The computational linguistic features adopted by today’s advanced AES systems cover grammar, wording, expression, fluency, and other aspects of essays. For example, some systems, such as e-rater (Burstein et al. 2013) provide an evaluation of grammar based on linguistic features; others assess the content based on features obtained through semantic analysis methods, such as content vector analysis (CVA) (Attali 2011) and latent semantic analysis (LSA; Landauer et al. 1998). However, to the best of our knowledge, very few state-of-the-art AES systems can automatically score the originality of essays, and very few studies have examined the relationship between the features of computational language used in existing AES and writing originality. Johnson et al. (2022) demonstrated that distributional semantic modeling has an impressive predictive power for predicting the extent to which a narrative connects divergent ideas. Ahmed and Feist (2021) used Linguistic Inquiry and Word Count (LIWC; Tausczik and Pennebaker 2010; Pennebaker et al. 2001) for automated creativity assessment of texts. Zedelius et al. (2019) used features from Coh-Metrix (Graesser et al. 2004) and LIWC to predict human scores of three sub-dimensions (image, voice, and originality) of writing creativity. Their results indicated that the computational linguistic features can be used to predict image and voice scores, but have a lower contribution in originality prediction.

When originality is defined as the uncommonness of the relative frequency of occurrence of plot lines of essay, it is determined not only by the intrinsic characteristics of the text, but also to a large extent on the reference group within which the essay has emerged (Silvia et al. 2008,  Zedelius et al. 2019). Namely, regardless of whether human scoring or automated scoring is used, it is necessary to develop a general perception of all the writing responses. Through the use of computational linguistic characteristics and semantic analysis, we can collect more evidence on the differences in language usage among essays.

### 1.2. Distributional Semantics in Creativity Assessment

The general idea of remoteness as a classic indicator of originality is very old (Wilson et al. 1953). Furthermore, according to the associational theory, novel ideas generally emerge from a combination of remotely related concepts (Mednick 1962). In recent years, researchers have been measuring creativity or originality using the distributional semantic representation based on the “distance”, e.g., connecting less relevant concepts into new concept combinations. To produce new combinations, extensive knowledge coupled with divergent thinking is required. Divergent thinking refers to the ability to produce multiple responses or solutions for a problem, contracting with convergent thinking in which there is only one correct solution (Guilford 1967). Psychologists believe that divergent thinking is the most important characteristic of creative thinking, and it is an indicator of creative potential (Dumas et al. 2020; Runco and Acar 2012). In contrast to divergent thinking, stereotyped semantic relations are associated with poor creative ability (Bendetowicz et al. 2018; Ovando-Tellez et al. 2019). In general, the more creative an individual is, the less constrained semantic association is (Bendetowicz et al. 2017; Benedek et al. 2017; Benedek et al. 2012; Kenett et al. 2014).

With the development of computational linguistics, computational methods have been applied to test the semantic relations between verbal expressions. Paulus et al. (1970) used stepwise regression approaches to predict originality based on a set of text mining features in divergent thinking tests. Forthmann and Doebler (2022) reviewed relevant studies in the past 50 years and acknowledged Paulus et al.’s (1970) value in the field of automated scoring of divergent thinking. Methods to measure creative thinking have the potential for further improvement. The key metric is the *semantic distance* calculated based on various distributed semantic models. Semantic distance based on LSA showed a negative correlation with originality scores of essays (Harbinson and Haarman 2014) and has a strong relation with creative cognition represented by single-word utterances (Prabhakaran et al. 2014). Yu et al. (2022) investigated the performance of maximum-associative-distance based on LSA for assessing response novelty in the ”Alternate Uses” task. “Given that the originality of a text can be defined as the degree to which an idea is semantically distant from other ideas, latent Dirichlet Allocation (LDA) (Blei 2012) is also used to compute semantic distances for measuring the similarity among texts (Shi et al. 2016) or to assess the novelty of ideas among texts on the website (Wang et al. 2019)”. LDA is a topic model algorithm based on a probability model, which can be used to identify latent topics in large-scale document sets; therefore, each document is viewed as a mixture of latent topics. Each document can be represented as a vector in which the elements are the probability that the document belongs to each topic. LDA introduces a Dirichlet distribution prior to document-topic distributions and uses Bayesian statistical learning algorithms to infer the topical structure of the corpus from the word co-occurrence frequency in a corpus. LDA estimation generates two outputs: A list of topics, with each topic represented as a vector of word distribution; and a list of documents, with each document represented as a vector of topic distribution. LDA model can be used to condense thousands of diverse text entries into a limited number of discrete topics (or subdomains) and simultaneously derive the latent topic structure for each text, which makes it particularly useful in learning the content of the text.

Another trend of language processing technology in creativity assessment is the use of pre-training models, such as word2vec (Mikolov et al. 2013b) and the Global Vectors for Word Representation (GloVe; Pennington et al. 2014), in obtaining the distributional semantics of essays to predict originality of the text. Furthermore, the automated scores generated through GloVe models can be used as an alternative to human scoring of originality (Dumas et al. 2020). Beaty and Johnson (2021) calculated five semantic distance variables on an open platform (called “SemDis”) and generated a latent semantic distance factor. The result shows that all five semantic distance variables and the compositional semantic distance have a strong correlation with human creativity and novelty scores of a series of creativity tasks. Johnson et al. (2022) used Google’s Bidirectional Encoder Representations from Transformers (BERT; Devlin et al. 2018) to generate context-dependent word embeddings to predict divergent semantic integration in writings. The current trend of automatic originality scoring comprises feature-based supervised approaches as well as other forms, such as large pre-training language models (Buczak et al. Forthcoming; Organisciak et al. 2022). These studies show that this field has scope for further improvement.

More recently, network science is increasingly used to quantify the strength of semantic associations. Several studies have indicated that semantic network (or graph) analysis is advantageous in creativity research (Kenett 2018; Kenett and Faust 2019). Most researches on the relationship between semantic network and creativity are conducted based on experiments of semantic divergent thinking. For example, participants are asked to think of three, five, or as many associative reactions as possible to a cue word in 1 min, and the shortest steps between each pair of words (or concepts) reflect the subjects’ perception of semantic similarity or dissimilarity (Landauer et al. 1998). Rossmann and Fink (2010) showed a positive correlation between creativity and associative distance in word pairs. Kenett et al. (2014) used the network science method to characterize the semantic memory structure differences of 96 clue words between high semantic-creativity groups and low semantic-creativity groups. Their results showed that the average shortest path length of high semantic-creativity groups was lower, while the degree of interconnection between adjacent points of a point on the graph was higher. Moreover, they found that the more subgraphs (or modules) in the network, the lower the level of creativity. The semantic network of highly creative individuals is more connected, flexible, and effective in diffusion (Kenett 2018; Bendetowicz et al. 2018), and these people build longer and distant associations through communication activation (Kenett and Austerweil 2016). The further the distance between concepts, the more creative the new combination will be (Kenett 2019). These findings suggest that highly creative people outperform less-creative people in perceiving connections between concepts, a point that also indicates a possible means of measuring originality in writing. Gray et al. (2019) quantified the extent of mind wandering “forward flow” within free association using a semantic network. Forthmann et al. (2022) demonstrated that the composites of semantic distance scores have excellent reliability across two types of creative thinking tasks.

However, most studies focused on the “Alternate Uses” task or “Creative Word Association” tasks, while other studies analyzed longer pieces of written text (Ahmed and Feist 2021; Forthmann et al. 2022; Johnson et al. 2022; Zedelius et al. 2019). These findings demonstrate the application of distributional semantic distance as a measure to predict creativity or originality as the semantic distance tends to have a strong negative correlation with originality in the context of word association tasks or open-ended prompts of writing tasks.

### 1.3. Essay as a Network for Automated Scoring

In the field of automated essay scoring, researchers used the network (or graph) features derived from an essay to predict the writing quality. Network-based features are used to predict the holistic score of the essay (Amancio et al. 2012; Antiqueira et al. 2007; Somasundaran et al. 2016) or another aspect of writing unrelated to creativity, such as fluency, development, etc. In general, the first step is mapping the essay into a network. Nodes in the network can represent words, phrases, concepts, sentences, or paragraphs of the text, while the edges reflect the relationships between nodes. The edge can be constructed based on semantic similarity, co-occurrence, or grammatical relationship of the semantic relationship between any two nodes. The network cannot only capture the words used, but also reflect the semantic association pattern of words (or concepts) in each text. The next step is developing diverse types of features based on the network to capture the characteristics of an essay’s network. The three common types of features are *Centrality, Path distance,* and *Similarity* in assessing writing.

*Centrality* has always been used in network analysis to estimate the importance of a node in networks. The methods for measuring centrality include Degree centrality, Closeness centrality, and Intermediate centrality. PageRank and Eigenvector centrality are two recent outstanding measures of central tendency in essay scoring (Somasundaran et al. 2016; Yang et al. 2022). *PageRank* (Brin and Page 1998) is used as a metric to simulate a “random surfer” on the network. The more central the node is, the easier it is to access; consequently, nodes with high centrality create link-intensive areas. For a given network, the higher the average network PageRank value, the higher the centrality, and it is often a negative correlation with essay development ideas (Somasundaran et al. 2016). *Eigenvector Centrality* is an alternative that takes into account the importance of the number of connections of a given node as well as its adjacent nodes. The centrality of a node’s eigenvector is proportional to the sum of the centrality scores of its neighboring nodes.

*Path distance* reflects various individuals’ cognition strengths (Gray et al. 2019; Kenett 2019). Path distance refers to the shortest path between two network nodes that can be reached through one or more edge connections. Students’ ability to organize words or concepts is influenced by semantic divergent thinking, a concept represented by the frequency of ideas between a given set of words. If a student can always connect words or concepts that are almost inaccessible in terms of semantic distance, it demonstrates their talent at constructing uncommon semantic connections on a network. Yang et al. (2022) combined word embedding and graph structure to represent students’ essays and explored the method of combining global and local semantic information to predict the writing quality of ideas. Ke et al. (2016) used complex networks to score Chinese essays and adopted in-/out-degrees, clustering coefficient, and dynamic network features.

*Similarity* is a more common metric in automated essay scoring history. It is used to quantify the degree of similarity between the target essay and the high-score essay or the reference text based on various natural language processing approaches, such as the CVA (Attali 2011), LSA (Cao and Yang 2007; Mikolov et al. 2013a), LDA (Kakkonen et al. 2008), and other distributed semantic models. When an essay is represented as a network, comparing the similarity between the two networks expands the method box under this metric. Yang et al. (2022) demonstrated that the closer the network of an essay is to the high-scoring essay, the more its content adheres to the writing prompt. In contrast, for original scoring, the main difference is that the goal is to measure the degree of difference between any two writings.

In the current study, we explored the semantic network drawn from an essay (hereinafter referred to as “semantic structure”) to capture more quantitative insights for predicting originality.

### 1.4. Limitations of Past Work

Computational linguistics provides an alternative to quantifying writing’s quality objectively. While using a topic model to represent an essay, the essay is represented as a series of latent topics, and the similarity of latent topic structure among essays can be used to assess the difference among essays. While using a network to represent an essay, compared to a distributional semantic representation of text, network analysis provides another fine-grained way to assess the difference based on the structured semantic association (Kenett 2019; Kumar et al. 2020).

One of the main limitations of distributional semantic models, such as LDA, LSA, and word2vec is that the word embeddings are context-independent. To fill this gap, Johnson et al. (2022) used BERT to generate context-dependent word embeddings depending on the sentences in which the words are used. However, the context stays at the level of “what words are used in the text”. Here, we hope to further refine the consideration of context dependence. Based on LDA, we divided a group of robust reference contexts in which the essay emerged for comparison. Based on the network representation of essay, in addition to considering the context in which each word appears, it focuses on how each word combines with other words to construct new relationship patterns. When an essay is mapped into a network, the significance and role of a specific word in different essays are very different, and the path between word pairs varies across essays, even if the context is composed of the same set of words. This inspires us to combine the semantic distribution and the network of the essay, and extracted features enlightened by previous studies, such as Kenett et al. (2014) to predict the originality of writing. Furthermore, in previous studies of creativity, although the semantic structures are represented as networks to compare differences, such as an individual’s semantic association distance in the word association experiment, researchers used to assign clue words and then require participants to make a judgment on the strength of the relationship between these words (Benedek et al. 2017; Kenett et al. 2014; Yee and Thompson-Schill 2016) or they controlled the choice of clue words based on computational methods (Bernard et al. 2019). However, in a particular writing task, students produced words (or concepts) by themselves often without any given clues, and then organized these words into a complete story or a meaningful discourse, which contains their own semantic structure.

To date, the automated scoring of essays based on networks is still in its early stage. The exploration of the relationship between the characteristics of the network of an essay and the quality of the essay is scattered, and most of them focus on the inherent linguistic characteristics of the text. There is no systematic study on the contribution of network structure to writing originality scoring. The current study was an attempt to narrow the gap between essay originality scoring and the network characteristics of writing based on distributional semantic theory and associative theory.

### 1.5. The Current Study

This study aims to explore a feasible path to predict writing originality by combining topic analysis and a semantic network. Our approach comprises two steps: In step 1, we use LDA estimation to identify latent topic structures in writings and then treat essays with similar topic generation probability as a reference group within which the originality of the essay will be meticulously evaluated. In step 2, the differences in the essays can be more finely examined within the reference group that belongs to a topic or a writing task. We evaluated originality prediction performance of a series of computational linguistic features that were developed through mapping essays into semantic networks.

In this study, we aim to address the following questions: (1) How should we quantify the reference group in regard to whose context an essay is going to be evaluated? (2) How should we develop quantitative computational linguistic features based on a semantic network to offer objective evidence for predicting writing originality?

## 2. Materials and Methods

### 2.1. Participants

The participants were eighth-graders from junior middle schools in China. Their ages range between 13 and 15, which is the general age range of Chinese eighth-grade students. They participated in a compulsory assessment of Chinese teaching and education quality monitoring test, which aimed to collect formative information about students’ knowledge of reading and writing. The last item in this test was a writing task in which each participant was randomly assigned one prompt out of three. The sample size of each prompt is presented in Table 1.

### 2.2. Materials

The semantic openness of the topics was different regardless of writing prompts and genres. In Prompt 1, students could easily understand the two keywords (“company” and “gift”), and describe, discuss, and express their feelings and past experiences. Prompt 2 had no notional word or entity; therefore, the semantic scope was broader than those of Prompt 1. Prompt 3 asked students to fill in the blanks in the title and then write an essay. As Prompt 3 required divergent thinking, it could be regarded as the most open prompt. We collected all of the students’ responses and graded the originality of their writing based on the originality rubric, which is introduced in detail later.

### 2.3. Rubric Scoring

A group of experts, including teachers, psychometricians, and language testing experts, developed a theoretical framework for scoring essay originality on a writing task for quality monitoring of a Chinese language test. A small random sample (15%) of essays from each task was extracted and examined by the experts. After several rounds of discussion and revision, a three-point originality scoring rubric was generated, and the descriptions of each level were compiled (see Appendix A). The rubric emphasized the degree to which ideas and content are novel compared to other essays under the same writing prompt.

Five raters (two male and three female master students of psychology and linguistics majors) were trained to score essays based on this rubric. Essays were graded as one, two, or three (corresponding to poor, medium, and excellent originality). Before the formal scoring, 10% of the sample essays were evaluated by the raters, and then any noticeable differences of opinion in scoring were discussed. After reaching a consensus on some differences of opinion, the raters scored all of the essays in a pseudo-random order independently. For each essay, the average score of the raters was used as the final originality score. Before the next step, nine essays were excluded: One blank answer, two copied sentences from other parts of the test prompts, one answer written in the non-Chinese language, five off-topic (off-topic means the essay does not contain prompt-adherence content and concepts, e.g., including a joke that is completely unrelated to the theme of “Company is the best gift”) or unrecognized slang (stacked idioms, slang, or unknown symbols that do not constitute smooth and meaningful sentences, often without punctuation). The average agreement measurement for the intra-class correlation in Tasks 1, 2, and 3 were 0.80, 0.79, and 0.78, respectively, which indicates good reliability according to Cicchetti (2001).

### 2.4. Research Tool

In step 1, we used the Python package Jieba (https://pypi.python.org/pypi/jieba, accessed on 3 January 2021) to perform pre-processing, such as Chinese word segmentation. We used the R package “stm” (Roberts et al. 2013) to implement topic analysis in step 1 in R 4.0.2. We pre-processed the Chinese corpus and constructed the network representation of the essays using Python 3. We extracted the features based on R package “igraph”. We used R package “glmnet” (Friedman et al. 2010) to model originality prediction and drew graphs using ggplot2.

### 2.5. Step 1: Topic Analysis of Essays

#### 2.5.1. Pre-Processing for Essays

Essay pre-processing was necessary as Chinese is written without spaces between successive characters and words. Each sentence was then transformed into word sequences by word segmentation, and stop words were dropped. The term “stop words” refers to common high-frequency words that possess little meaning. For example, function words (such as auxiliary words, modal particles, and modal verbs) were dropped to make the model focus on ideas and content to the greatest extent possible since previous studies suggest that function words may underestimate semantic distance even if the core response idea is highly original (Dumas et al. 2020). We used Jieba, a Python package that can deal with Chinese words, and then built a corpus of Chinese essays for each writing prompt. The number of unique words from Prompt 1 was 3897, the average length of the essay was 52.275, and the standard deviation was 15.029; for Prompt 2, the corresponding numbers were 4173, 50.632, and 14.552, respectively, and in Prompt 3, the numbers were 4228, 51.617, and 15.546, respectively. The document-term matrix obtained from each essay was used to estimate the LDA, and the words and their relationships in the sentences constructed the network that has been illustrated in step 2.

#### 2.5.2. Topic Analysis

We used the LDA topic model to estimate the latent topic of essays for classification. LDA is particularly useful since it does not require researchers to specify the topic structures in advance; rather, it uses modeling assumptions and text attributes to generate a set of topics and estimate the probability of writing on each topic. We used each writing sample as a single document unit for training LDA models. This process involves two steps: (a) Determining the number of topics, and (b) using the determined LDA model to estimate the probability of a particular essay being generated based on the topic, and the probability of which topic will generate any given text.

The topic can be regarded as a latent variable, which is estimated based on the probability of word co-occurrence (Mohr and Bogdanov 2013). LDA estimates two probability matrices simultaneously: *γ* matrix reports the probability of each essay topic; *β* matrix reports the estimated probability that each word arises from a given topic, and both distributions are subject to the Dirichlet distribution. Dirichlet distribution, also known as multivariate beta distribution, is a type of high-dimensional continuous distribution with standard simplex as support set in the field of real numbers. It is a generalization of beta distribution in the high-dimensional case. Our goal was to estimate the latent topic structure of each essay and the distribution of words in each topic. Specifically, we assumed that the topic number is *K*, and the estimation of all probability distributions is based on *K* topics.

Similar to exploratory factor analysis, topic modeling also has difficulty in determining the number of topics (subdomains) in the corpus in advance. In this study, we combined the diagnostic statistical indicators of the model with the subjective judgment of experts to determine the number of topics. According to the number of essays and the experience of the experts’ ratings, we first calculated the diagnostic statistics of a series of LDA models with each writing topic task numbered from two to ten. From a statistical point of view, we relied on Roberts et al.’s (2014) suggestion to use *semantic coherence* (Mimno et al. 2011) and *exclusivity* (Airoldi and Bischof 2012) as complementary indicators for this model. *Semantic coherence* refers to the overall semantic consistency of popular words in a topic. *Exclusivity* summarizes the semantic exclusivity of a topic relative to other topics (Roberts et al. 2014). We chose the ones with high consistency and exclusivity as candidate models. Then, three human coders who participated in the originality scoring in this study were asked to evaluate whether the essays with high load had clear topic meaning. Through this, the optimal number of candidate topics and models were determined. Based on the representative words in each topic generated by the optimal model and their β values, the human coders labeled topics with substantive meaning. We used an R package “stm” (Roberts et al. 2013) to implement topic modeling and set the model to run 500 EM iterations at most, set the convergence tolerance to 0.00001, and set other parameters with default options, e.g., a uniform topic prior to alpha defaulted to 50/*K*, which is the prevalence hyperparameter in collapsed Gibbs sampling in LDA initializations.

Topic analysis categorized the essays into a series of topics, which provide more refined reference groups for evaluating originality in step 2. In the result section, we reported the diagnostic statistics and the substantive labels from the optimal model.

### 2.6. Originality Prediction Based on Networks

The relationship between originality and semantic networks is always specific in a manner that is clearly demonstrated by the examples mentioned in the introduction. In this section, we mapped each essay into a network in which nodes and edges carry quantitative semantic information. Then, we developed a group of features based on the network characteristics to predict writing originality, especially within a topical context.

#### 2.6.1. Essay as a Network

Based on the pre-processed corpus, each essay was represented as an *N* × *N* symmetric matrix. *N* is the number of unique words in an essay, and the labels of row-names and column-names are the unique (non-repeated) words. Each matrix cell represented the co-occurrence relationship of the corresponding word pairs. If a pair of words appeared in the same sentence, the value of the cell was 1; otherwise, it was 0. It is noted that although only unique words are retained, if the word appears in distinct sentences, for example, in four sentences, all the different co-occurrence relationships of the word in these four sentences will be preserved. What is ignored here is the information about the relationships that occur multiple times between the same word pairs. Based on this matrix, an unweighted undirected network was obtained. For each essay, unique words were mapped to a node on the network and an edge between nodes corresponded to a pair of words with a co-occurrence relationship. Edges were not weighted since the current research focuses on checking the network structure and converting them into a unified weight (=1.0), in order that the shortest path between the nodes in the network can be calculated.

Each node in the network represents a word or concept in the essay. There are two types of distances between each pair of nodes: One is the closeness between the natural semantics of the words represented by the nodes; namely, the semantic distance; the other is the number of steps that a pair of nodes goes through in the network; namely, the path distance. Then, we extracted features based on these two types of distances and other characteristics of the network.

#### 2.6.2. Network-Based Features

1. Feature extraction based on semantic distance

We utilized the pre-trained word embeddings of word2vec (Mikolov et al. 2013b) to serve as the quantitative content semantics of the nodes. Previous studies show that word2vec preserves real semantic relationships between words more effectively than LSA and other models (Mikolov et al. 2013a), and may quantify originality better than LSA (Dumas et al. 2020). In this research, each word (or concept) was represented as an 800-dimensional vector, the semantic similarity between words was obtained by calculating the cosine similarity between vectors, and the value was in the range of 0–1. The word embeddings in this study were derived from the five-million-word Baidu Encyclopedia training corpus. This research assumed that semantic distance based on word2vec reflects the semantic intimacy between words from a human being’s perspective.

Semantic distance is equal to a value of 1 minus the cosine angle of two words’ embeddings. After normalization, the value was in the range of 0–1. The closer to a value of 1, the farther the semantic distance is between the two words. We calculated three features based on semantic distance: (1) The average semantic distance between two adjacent nodes (*w2vmean*); (2) The maximum semantic distance between any two nodes (*w2vmax*), similar to “forward flow” in Gray et al. (2019) and “maximum associative distance” in Yu et al. (2022); (3) The sum of semantic distances of all nodes (*w2vsum*). When considering writing as a divergent thinking task, the longer the semantic distance of the words, the less likely the words or concepts are used together in general. This process is conducted on each essay separately; therefore, each essay receives a *w2vmean*, *w2vecsum*, and *w2vecmax* measure.

2. Feature extraction based on path distance

In our study, we developed five path distance features: (1) The number of shortest path distance between nodes, i.e., one divided by the number of nodes (*Path 1*); (2) the proportion of the number of the shortest path distance between nodes, i.e., one to the total number of paths (*P1dpn*); (3) the average number of path distances in the network (*Pathmean*); (4) the maximum path distance in the network (*Pathmax*); (5) the sum of the path distances in the network (*Pathsum*). These features are similar to the statistics in Kenett’s previous studies, i.e., *Pathmax and Pathmean* are comparable to *D* (*diameter*) and *ASPL* (*average shortest path length*) in Kenett et al. (2014).

It should be noted that directly connecting words with close semantic distance does not necessarily indicate a higher level of creativity than indirectly connecting nodes with far semantic distance. It is reasonable, in the case of an essay with a large number of words, to comprehensively consider semantic distance and path distance to evaluate the development of words or concepts in the essay. Here, we proposed a feature combining semantic distance and path distance (*V2bp1*); it is calculated by the ratio of semantic distance to the sum of the shortest path in the network, which reflects the average semantic change of each path step in the word sets generated by writings.

3. Feature extraction based on centrality

We used two types of features, *PageRank and Eigenvector Centrality,* to measure centrality. The centrality features are called *CC* (the networks clustering coefficient) statistic, which refers to the probability that two neighbors of a node will themselves be neighbors (Kenett et al. 2014). In our study, it was assumed that the more scattered the links in that network, the more divergent the concepts or ideas were in the essay. This decentralization showed that the author emphasized the detailed development of multiple concepts rather than repeating a single word or concept. Then, we took the negative logarithms of these two types of measurements as the features: The mean of PageRank of the network (*Logprmean*), the max PageRank (*Logprmax*), the sum of the PageRank (*Logprsum*), and the mean of Eigenvector Centrality (*Logevcnmean*). The higher the degree of network decentralization, the higher the value of these two features and the more scattered the links in the network, which indicates a higher likeliness to develop uncommon ideas.

4. Features based on similarity

We evaluated the similarity of networks from two aspects: One is the similarity of nodes, which aimed to examine the extent to which words or concepts used in an essay overlap with other essays, including “the maximum number of the same nodes” (*Intervecmax*) and “the standard deviation of the same number of nodes” (*Intervecsd*). The *Intervecmax* of a particular essay is the maximum number of identical nodes obtained by comparing the essay with all other essay networks, and, in turn, the *Intervecsd* of a particular essay is the standard deviation of a group of the number of same nodes obtained by comparing a specific composition with all other composition networks.

Furthermore, we compared the similarity of essay semantic structures by calculating the correlation of network structures, which considers the similarity of nodes as well as edges. It can be interpreted that even if a similar group of words is used, variations in organization and connection patterns are likely to form a new semantic structure, which is likely to indicate that the essay has original ideas. Here, we used the “sna” package (Butts 2008) of R to calculate the product-moment correlation of the adjacency matrix (Butts and Carley 2001) of two networks to represent the similarity of networks.

In particular, based on the different contexts in which the essay is compared, we calculated two features of network similarities: One is the correlation between the essay and other essays for a given topic (*topicgcor*); the second is the network correlation between the essay and all other essays for a given writing task (*taskgcor*). The calculation method of *topicgcor* and *taskgcor* is similar to the “association correlation networks” proposed in (Kenett et al. 2014), i.e., Pearson’s correlations between the two word association profiles represented by two matrices.

#### 2.6.3. Essay Score Prediction and Calculation

We used network-based features as predictors to generate the score of originality by lasso regression (Tibshirani 1996). Lasso was performed since (1) some essay topics fail to meet the required minimum sample size for regression analyses (Harris 2001), and (2) lasso analysis is suitable for feature selection with collinearity predictors. We trained the lasso models for each writing prompt using all the essays written about that task, which was called the “task-level model,” and trained the models for each topic that used the essays with the highest probability on this topic, called the “topic-level model.” We used the function “cv.Glmnet()” in R package “glmnet” (Friedman et al. 2010) for lasso modeling, and Leave-One-Out cross-validation was used to increase the methodological quality.

## 3. Results

In this section, we will first present the results of topic analysis, and then share the performance of originality prediction of network-based features used in the topic-level and task-level models.

### 3.1. Number of Topics and Substantive Labels

**Figure 1:**
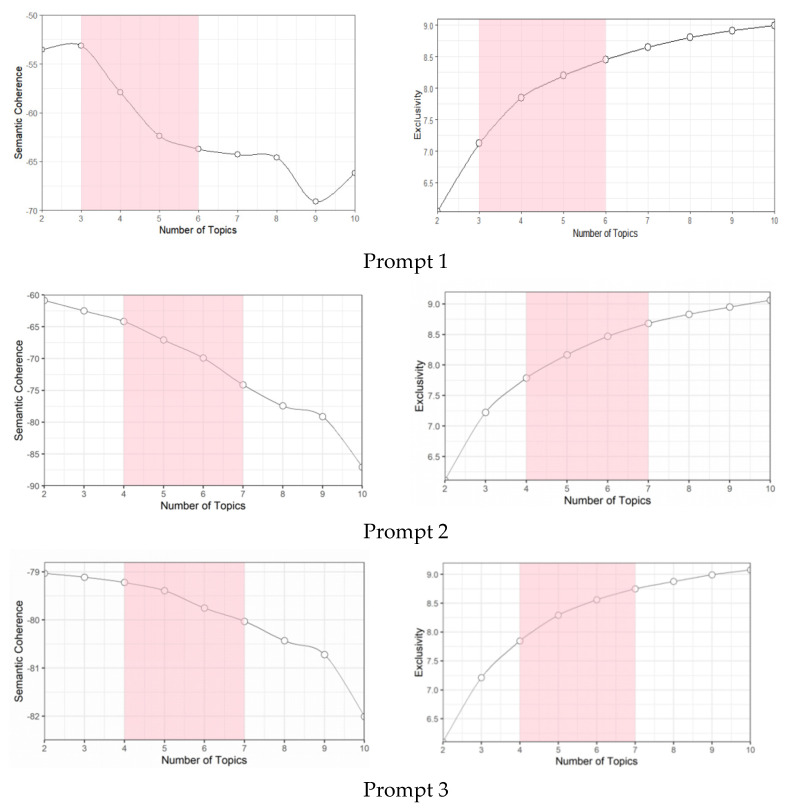
Optimal topic selection. A set of the number of topics is displayed according to two semantic coherence and exclusive diagnostic statistics. The shaded rectangle displays the range in which topics were evaluated for substantive meaning.

LDA analysis requires researchers to specify the number of topics in advance. As mentioned above, we started the LDA modeling process by specifying two to ten topics, and then used two diagnostic indicators for semantic coherence and exclusivity to narrow the selection range of topic numbers. Figure 1 shows the change in the two indicators across topics. In the graph of semantic coherence, the *y*-axis indicates the co-occurrence probability of the top words in the topic, and a value close to zero indicates that the words tend to appear more frequently at the same time, while a larger negative value indicates the opposite. In the exclusivity graph, the higher the value on the *y*-axis, the better the performance of the model in separating topics. The results showed that when the topic number is two and three, Task 1 had the highest semantic coherence, but the lowest exclusivity. The semantic coherence for topics three to six gradually decreased with the increase in the number of topics. The semantic coherence for topics seven to ten was the worst, and the increase in exclusivity increase reduced. The semantic coherence for topics two to seven in Task 2 decreased with the increase in the number of topics, and the models with more than seven topics showed a faster decreasing trend. In contrast, the models with fewer than four topics had lower exclusivity. The semantic coherence for Task 3 with two to seven topics changed a little, and the exclusivity of the model with more than four topics was higher. As topics with both cohesion and exclusivity are likely to be semantically useful (Roberts et al. 2014), semantic coherence, as well as exclusivity, need to be taken into account. Based on these results, we further evaluated that the topic numbers of Task 1 are three to six, and Task 2 and Task 3 are four to seven to balance the two indicators within an acceptable range.

The diagnostic indicators of the model provide general guidance for the selection of the model but do not directly reflect the substantive meaning of the topic identified by the model. To evaluate the meaning of topics, three experts, who participated in the scoring, further reviewed the content validity of the topics generated by all the candidate models. Specifically, the experts assigned substantive labels to each topic by reviewing the high-frequency words for each topic. They independently evaluated the substantive meaning of various topic model solutions and then reached a final consensus through discussion. In this process, some high-frequency words appeared in more than one topic. For example, in Prompt 1, the word “companion” was a high-frequency topic word that provides less information to distinguish the thematic meaning. To provide more information for the representation of topics, we also provided high-frequency exclusive words (Airoldi and Bischof 2012) for experts as additional references. Finally, the topic solutions of the three writing prompts were determined by experts. The optimal model for Prompt 1 was five topics (coherence = −59.546, exclusion = 8.182); Prompt 2 was six topics (coherence = −62.390, exclusion = 8.197), and Prompt 3 was six topics (coherence = −79.753, exclusion = 8.560). When adopting the optimal model, each topic had substantive meaning and generated the latent topic structure for each essay. Table 2 lists the substantive description of each topic and the proportion of essays with the highest topic probability.

**Figure 2:**
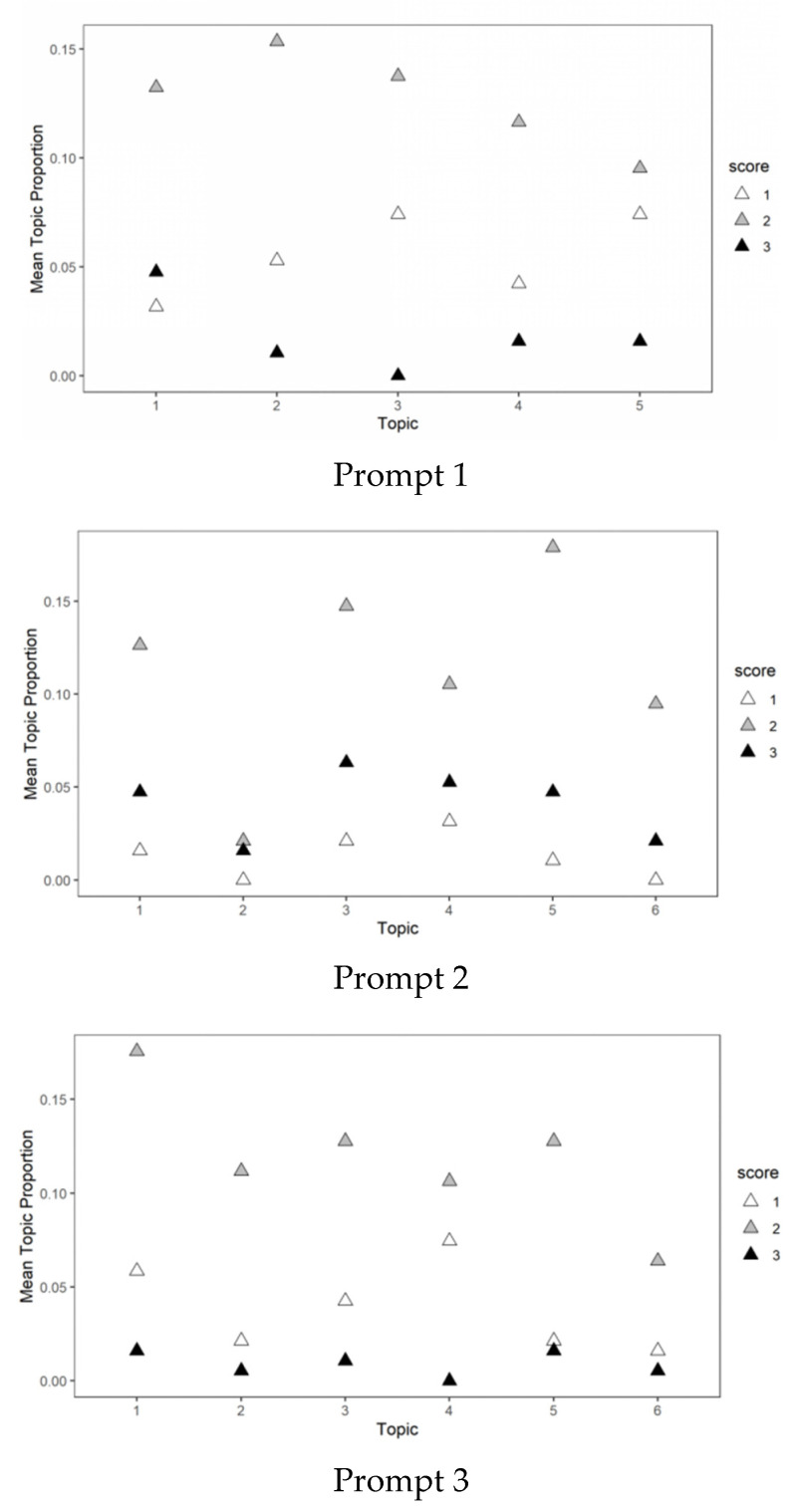
Topic proportions over scores. The horizontal axis represents the topics in a given prompt; the corresponding labels of topics are included in Table 2.

Figure 2 shows that most of the topics contained high-scoring essays as well as low-scoring essays. The topic model is a bag of words model generally used in text classification to represent documents as vectors. For any text, once the word order, grammar, and syntax are ignored, this representation is regarded as an arbitrary collection of words. Essays belonging to the same topic estimated by LDA only indicated the probability of having similar word use. Our data showed that essays with varying originality levels belonging to the same topic, and the distribution of originality scores on different topics was also inconsistent. When an essay is clustered into a specific topic with similar patterns of vocabulary usage, the originality of the essay is expected to be reflected in the distinctive creation of word connections and distance. In the next part, we further explored the relationship between the network characteristics for semantic structure based on the context derived from topic analysis. We compared how an essay, in a quantitative and fine-grained reference group, is different from others.

### 3.2. Semantic Structure Based on Network and the Human-Rated Score of Originality

**Figure 3:**
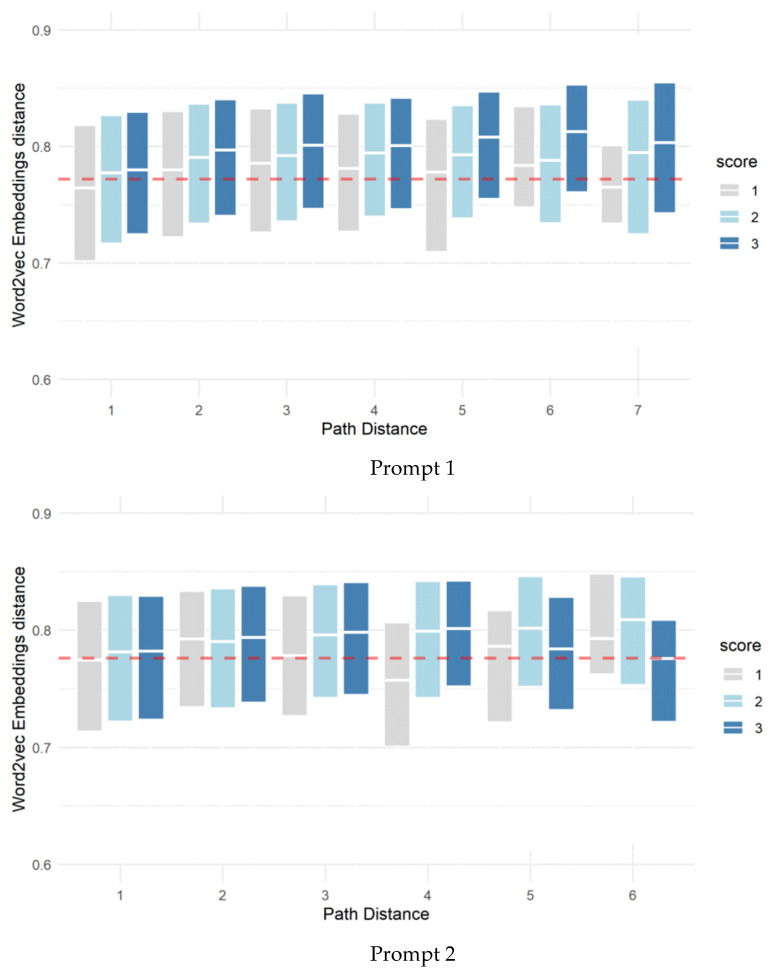

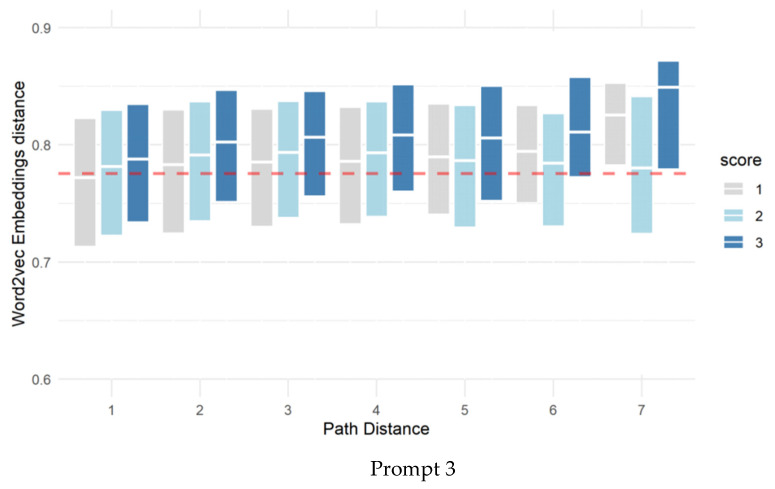
Semantic distance and path distance distribution cross over originality scores depicted at the level of essays. The *x*-axis indicates path distance, while the *y*-axis indicates the semantic distance calculated based on word2vec embeddings. The bands correspond to the inference representing 95% of the density interval for the given score point. The horizontal lines in the boxes indicate the mean semantic distance between the nodes for the given score point.

The change in semantic structure is mainly reflected by network differences, which are measured by the node’s semantic and path distances between any two accessible nodes. There were significant differences in the average semantic distance between essays with different human scores (Task 1: *d*_2-1_ = 0.15, *p* < 0.001, *d*_3-1_ = 0.28, *p* < 0.001, *d*_3-2_ = 0.12, *p* < 0.001; Task 2: *d*_2-1_ = 0.04, *p* < 0.001, *d*_3-1_ = 0.12, *p* < 0.001, *d*_3-2_ = 0.03, *p* < 0.001; Task 3: *d*_2-1_ = 0.10, *p* < 0.001, *d*_3-1_ = 0.29, *p* < 0.001, *d*_3-2_ = 0.19, *p* < 0.001, where *d* refers to Cohen’s (1988) and the subscript of *d* indicates which two score points of essays are compared). This indicated that originality scores improve with the increase in semantic distance, which is similar to the results of previous studies. Figure 3 shows that the longer the path distance, the higher the difference in semantic distance between high-scoring essays (three points) and other essays (one and two points). For example, considering the difference in semantic distance between essays with different scores when the path distance was 7, which is more apparent than other path distances, the variations of the semantic distance across different scores of essays were as follows: Task 1: *d*_3-1_ = 0.59, *p* = 0.002, *d*_3-2_ = 0.43, *p* = 0.005; Task 2: *d*_3-1_ = 0.27, *p* = 0.419, *d*_3-2_ = 0.93, *p* < 0.001; while a few long paths with opposite patterns were as follows, such as Task 3: *d*_2-1_ = −0.29, *p* = 0.002, *d*_3-1_ = 0.31, *p* = 0.003, *d*_3-2_ = 0.60, *p* < 0.001, where *d* refers to Cohen’s (1988) and the subscript of *d* indicates which two score points of essays are compared. The overall trend showed that the longer semantic or path distance is likely related to higher originality. Furthermore, in our study, originality is defined as a highly contextual trait, which needs to be compared to the “difference degree” of semantic structure among the essays to be evaluated together. For this reason, we need to situate the relative predictive performance of these features in a different reference context.

### 3.3. Examples of Essay Networks with Different Originality

In addition to the general statistics of the overall sample reported above, we randomly selected three essays with different originality scores as an instance to illustrate the relationship between semantic distance and path distance in detail. Furthermore, we created a visual network display of the essays for subtle observation, including the appearance of the network and the distance changes between similar nodes.

**Figure 4:**
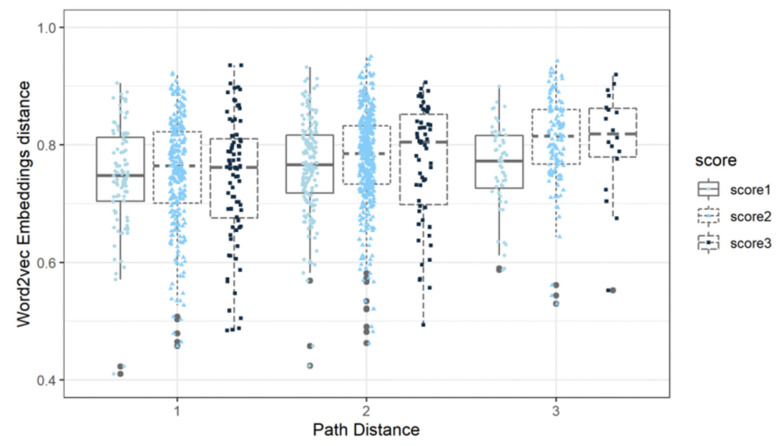
Boxplot for semantic distance and path distance for the given score point; bands corresponding to the inference represent 95% of the density interval. The whiskers on the boxes indicate variability outside of the upper and lower quartiles, and the horizontal lines in the boxes indicate the mean semantic distance for the given score point. The distance of each pair of nodes for a given essay is dotted against the x- and y-axes.

Three random Prompt 1 essays with different human scoring of originality (a one-point and two-point essay from Topic 2, and a three-point essay from Topic 5) were used as examples. These three essays all contained the high-frequency words “parents” and “gift.” Figure 4 illustrated the semantic distance and path distance of the three essays. It shows that the semantic distance of the three-point essay with a path distance of two was higher than the one-point essay, but the difference between the semantic distance of the three-point essay and the two-point essay was not very clear.

**Figure 5:**
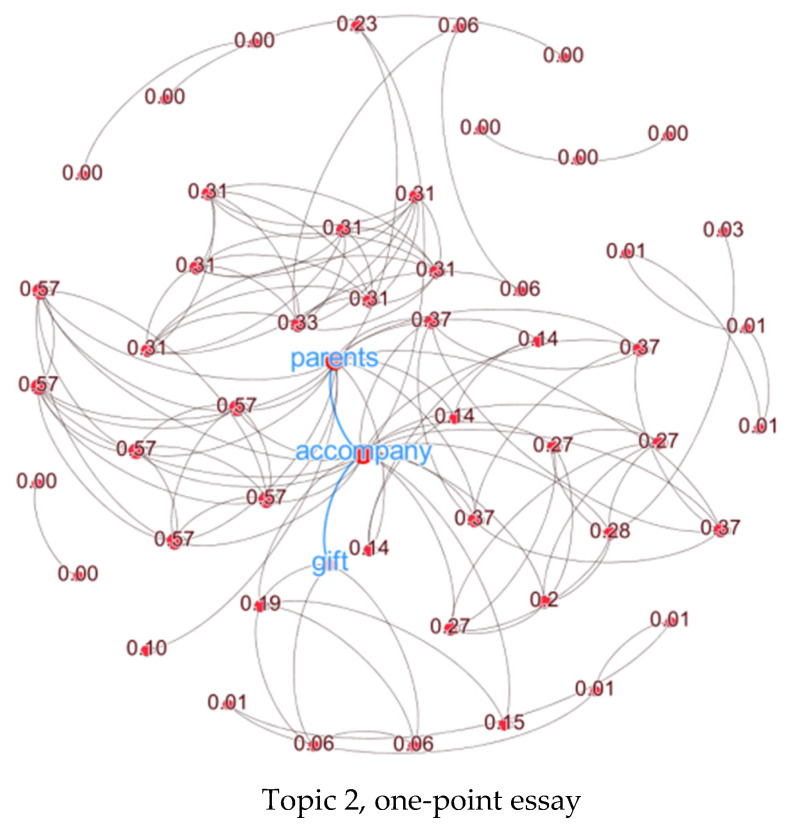

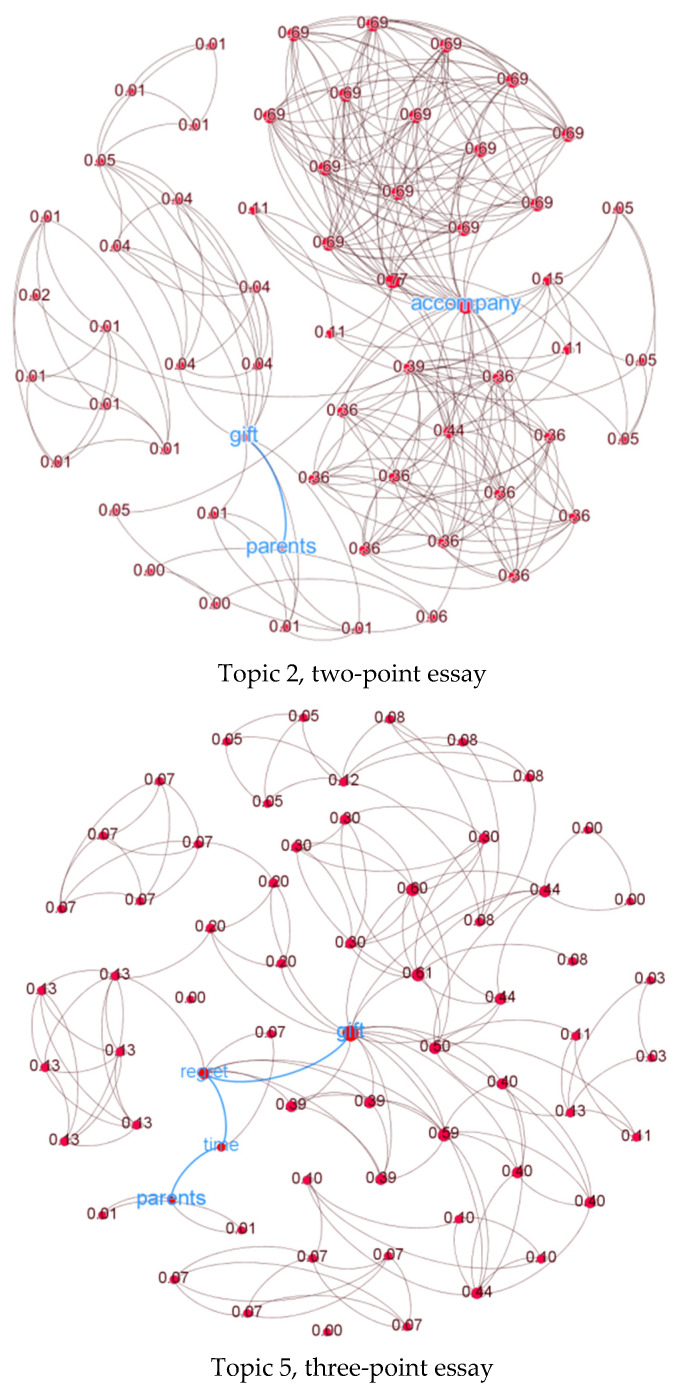
Examples of individual semantic networks for one-point, two-point, and three-point essays. The nodes represent single words and are labeled as numbers (normalization of Logevcnmean); the larger the node size, the higher the centrality. The gray lines represent the edges connecting the nodes.

Figure 5 visualizes the networks of the three essays. The size of the node is the normalization of the node’s eigenvector centrality, and the larger the node is, the stronger its centrality. There are many nodes on the boundary of the one-point essay network with only one or two neighbors, which form many paths without connected triangles. The node with the highest centrality is “companion”, and the node “parents” and “gifts” are connected with the node “companion” only through one side. For the two-point network, there are more triangles and two subgraphs with dense connections, which indicates that these nodes can be connected through a shorter path, and the links between these two subgraphs and other parts of the network are fewer. The central node is also “companion”, but the connection between “parents” and “gifts” is farther away from the center. For the three-point network, writing about parents working long hours, the author was frustrated even if they received a gift. There are almost no unclosed paths where the nodes and edges are distributed and scattered. The center node is “gift”, and the path distance between “gift” and “parents” is three, which is longer than the other two.

### 3.4. Originality Predicting and Features Contribution

Using network-based features as predictors, lasso regression was performed on each topic and writing task separately. We chose the lambda value that minimizes the mean-squared error to model; therefore, the independent variables were selected according to their importance and considering the collinearity between them. The results showed that for the task-level model, the total variance of the three task models was between 19.7% and 27.1%, and the variance of most topic-level models is higher than the corresponding task-level models. The total variance of the topic-level models in Prompt 1, Prompt 2, and Prompt 3 was between 24.9% and 64.1%, 29.7% and 68.6%, and 18.8% and 63.5%, respectively. The results of each task-level model and topic-level model are presented in Table 3.

**Figure 6:**
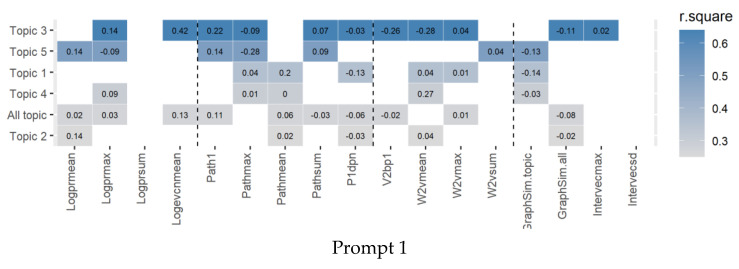

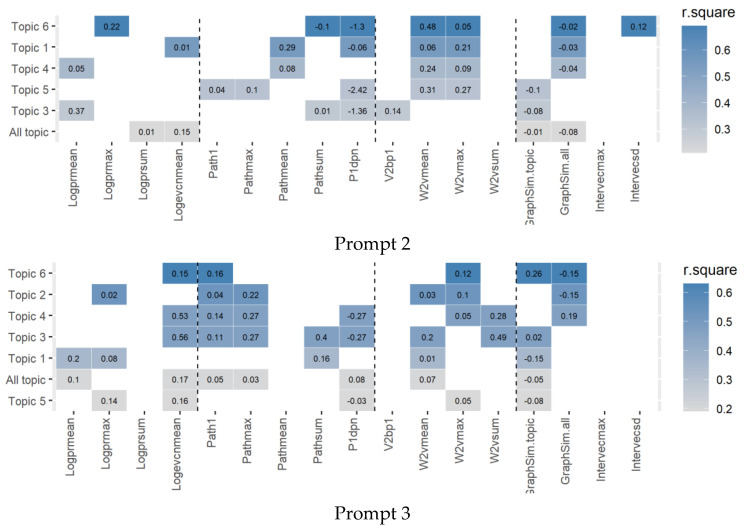
Heatmap of the R-square values of lasso. Colored cells display the selected features for predicting originality scores for each topic. The darker the cell, the higher the R-square in the prediction model. The three vertical dashed lines separating four types of features from left to right are centrality, path distance, semantic differences, and similarity. The “All topic” line of each panel is the result of task-level lasso analysis, which takes all essays under the given task for modeling; the other lines are the results of topic-level models using essays under the corresponding topic.

Figure 6 shows the feature selection by lasso analysis. For originality, topics in the same writing task present different feature solutions, the semantic distance features, path distance features, centrality features, and similarity features were often used in combination, and the task-level model is less effective than most of the corresponding topic-level models. Most of the features have consistent direction-crossing models. The negative logarithm for the centrality features (Logprmean, Logprmax, Logprsum, and Evcnmean) consistently predicted the originality positively, which indicates that the more the decentralization, the more ideas develop together and the higher the essay originality. Most of the features based on path and semantics distance positively predicted originality, which indicates that the longer the semantics and path distance, the higher the originality score. The feature P1ben negatively predicted originality, which indicates that the more concepts that can be directly connected in the network, the lower the originality tends to be. Intervecsd positively predicted originality in Task 2, indicating that the more unique the nodes are compared to the other essays, the higher the originality of essays. However, it is worth noting that Intervecmax also positively predicted originality, which is inconsistent with common sense. Relatively, GraphSim.topic and GraphSim.all negatively predicted originality in most topics as we expected, with the exception of Topic 3 and Topic 6 in Prompt 3. Based on the performance difference between these two types of similarity features, the high originality of essays may not only be the result of using unusual words, but also due to the fact that novel relationships between words (or concepts) are constructed.

**Figure 7:**
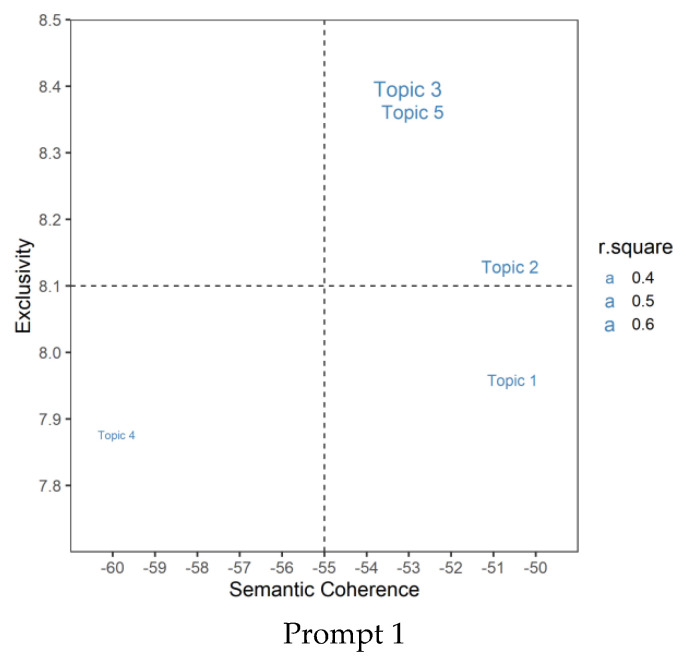

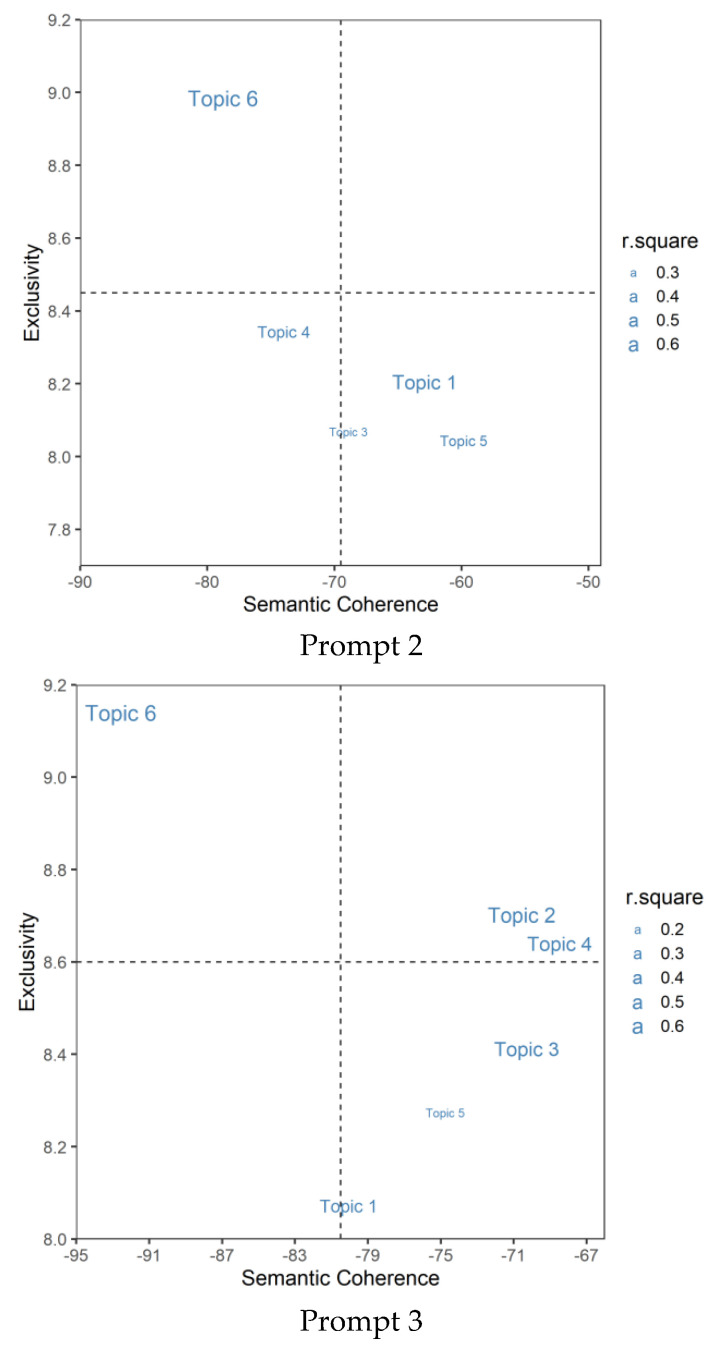
Topic quality and R-square. The quality of the topic is divided into four quadrants based on two diagnostic statistics (semantic coherence and exclusivity), and the size of the topic label reflects the R-square of the originality prediction for the given topic.

Figure 7 shows the relationship between topic quality and the R-square in each topic-level model. We found that the topic-level models with higher R-square are mostly located in the upper two quadrants, while the models with lower interpretation rates are located in the bottom two quadrants. It seems that the higher the exclusivity of the topic, the better the prediction performance of the automated scoring. In other words, the higher the quality of a topic obtained in the first step of the topic analysis, the more likely it is to be conducive to the prediction of the originality by its topic-level model.

## 4. Discussion

The goal of this research is to integrate topic analysis with semantic networks to predict the originality of essays. The results show that the four category features that combined distributional semantics and network characteristics contribute significantly toward predicting essay originality, especially in the topic-level models based on LDA.

### 4.1. Moving beyond Distributional Semantics for Originality Scoring

The results show that the farther the semantic distance, the more the contribution to originality, which is consistent with associate theory (Mednick 1962; Wilson et al. 1953) and previous studies based on computational linguistic features (Beaty et al. 2016; Dumas et al. 2020). However, we have further findings beyond the separate analysis of semantic distance. Using LDA to analyze the latent topic structure of writings, we can quickly understand a corpus that human raters are not capable of. The results validate the role of LDA in constructing an effective quantitative context for assessing the originality of writings. Compared to using LSA distance or a latent topic structure for assessing the similarity of writing creativity (Attali 2011; Cao and Yang 2007, Kakkonen et al. 2008), in our data, there is no positive straightforward relationship between relative topic frequencies and originality, each topic contains high- and low-level essays and the distribution of originality scores varies across topics. This indicates that the level of originality of the two essays will still be different within the same topic, which is likely caused by the different word relationship patterns in the two individual essays.

Furthermore, previous studies have reported on the relationship between creativity and divergent thinking (Kenett and Faust 2019; Silvia et al. 2008), which indicated that highly creative individuals can often build unexpected connections between concepts (Bernard et al. 2019; Rossmann and Fink 2010). However, it has not been tested in the essay scoring. The most apparent difference is that the edges in these studies mostly represent the semantic relationships between nodes and ignore the context. The edges between nodes in the current study are changing across the networks, which can reflect the unique context of each essay. The current study proved that the network representation of the writing provides a more refined method to quantify the global differences in the relationship patterns between words, similar to previous studies (Bendetowicz et al. 2018; Kenett 2018, 2019; Yu et al. 2022). Furthermore, this study indicates that the originality of an essay not only depends on the connection pattern between nodes in the network, which vary from essay to essay, but also on the semantic similarity between two words, which is calculated based on the encyclopedia knowledge corpus that is consistent in all essays. Students with a high level of originality in writing are often able to generate original ideas and build new connections between words or concepts that are far away in semantics. In our study, the features based on semantic distance and the features based on path distance both have positive predictive effects on originality. This indicates potentially far-reaching and important implications for combining the distribution semantic theory and network analysis for automated scoring of originality.

There is currently no standardized way for automated writing originality scoring. In the past, most research on the automatic scoring of essays based on networks aimed to predict the holistic score or the aspects unrelated to originality. Network-based features show excellent performance in evaluating writing quality (Amancio et al. 2012; Antiqueira et al. 2007; Somasundaran et al. 2016; Yang et al. 2022). However, the theory and operation for automated scoring of writing originality using computational linguistic features are far from enough to cope with practice. The result of this study indicates that the integration of distribution semantic and network analysis has substantial potential in automated scoring for writing originality. By systematically analyzing the functions of network-based features, we move forward to develop a reliable and automated approach to link the creativity theory to the automated scoring of originality.

### 4.2. Insights for Human Scoring Based on Feature Analysis

Ideally, the originality of the essay has to be assessed in the context of the reference group in the writing task. However, human raters find it difficult to assess the degree of difference among essays and struggle in the early period of rating owing to the lack of familiarity with the context. Even with scoring rubrics and training, the implicit criteria for the degree of “difference” in human raters will be inconsistent since they might not have the same perception of things, such as the degree of novelty, concepts, or ideas.

The results of feature analysis in this study can provide some objective evidence and insights for human scoring.

Some statistics and visualization can be given to human raters to support and facilitate their rating process. a) Metrics based on semantic distance (e.g., w2vmean) provide the human rater with quantitative perception about the degree of semantic divergence of writing. For example, in Prompt 1 (“company is the best gift”), “company” and “gift” are notional words with clear meanings; in Prompt 2 (if we do it again), there are no substantive words in the title, which gives students more space to develop their thinking; Prompt 3, i.e., “I forgot“, is an incomplete title and needs to be supplemented; therefore, Prompt 3 sustains a more open writing space than Prompts 1 and 2. The semantic distance between the words supplemented by students and “I forgot” offers a straightforward and stable value to represent the semantic relationship between the two parts. b) Metrics based on similarity (e.g., *intervecmax, graphSim.topic, and graphSim.all*) illustrate the extent to which words and word relationships in an essay are different from other essays. *Intermax* represents node similarity features and has positive contributions for predicting, thus implying that the more words (or concepts) in a particular essay reoccur in other essays, the higher the originality of the essay. In addition to statistical indicators, this approach can provide human raters with a visual network of essays to help them quickly understand and grasp the global semantic distribution of an essay. For example, the visual network shows whether the essay has a prominent center, whether the connections on the network are dense and evenly distributed, whether there are any apparent isolated modules, and whether the main body of the essay is far away from the central words. It should be noted that, at present, this information is only one of the auxiliary sources for originality scoring and cannot be used as the only criterion for evaluation.

Based on the empirical findings presented in the current study, it can be concluded for human raters that: (1) The criteria for judging originality across different topics are variable. The weight for the prediction of features on different topics varies. Therefore, human raters may focus on different aspects of writing when the reference group is changing. (2) Glimpsing or scanning through the words in an essay is likely to lead to missing the novel ideas that are written in simple and plain language. The current research shows that, for the same word set, unusual connections (or path distance) among the words will increase the originality of essay. (3) Topic relevance is still a fundamental quality to be assessed. The title of the essay given in the writing task limits the semantic divergence, which means that students should not be significantly “divergent”. It can be explained that the feature is highly related to the essay’s relevance to the topic. Namely, an original essay cannot deviate from the requirement of the writing task. When evaluating essay originality, human raters also have to judge whether an essay is relevant (or appropriate) to the title. We argue that originality is a point on the spectrum, sliding between “relevance” and “divergence”. When assessing open writing tasks, human raters must make a judgment regarding the trade-off between originality and straying off-topic.

### 4.3. Limitation

This study has certain limitations. First, the results of topic analysis by the LDA model will affect the performance of originality prediction. It should be noted that the value of topic modeling is to assess originality within topical contexts, which show a better fit in Table 3. In practice, if a new topic is introduced into the writing task, it is better to retrain the topic models to generate new reference contexts. However, retraining is not a necessary condition for using this method. As we know, the new topic or highly original essay has not been dropped from the process of scoring. When an essay belongs to a new theme, it means that the words used in the essay are unusual. Although it may be classified as an inappropriate topic, its unusual words can still be captured within any topic based on the node similarity features. However, when many essays are assigned to be graded, we suggest retraining the LDA model to improve the quality of the method.

We further address the significance and practicalities, which is also reflected in the sample size of each topic generated by LDA. In our data, lasso regression is modeled according to a feature’s importance in score prediction. As the sample size was limited on Topic 2 in Prompt 2, topic-level modeling cannot be performed. Moreover, we recognize the potential problems of network-based features. Whether the semantic distance calculated based on word2vec perfectly corresponds to the human rater’s understanding needs to be further explored.

Second, writers can “deceive” the AES systems by using unusual but meaningless ideas collocation. For example, it is possible to increase the semantic distance by deliberately using random words in order that the semantic-based features will fail to provide valid diagnostic semantic “differences”. In practical applications, “off-topic” or “nonsense” essays should be removed before automated scoring or exploring new technology, wherein the two processes can be implemented simultaneously.

Third, this research does not intend to prove that the features we extracted are the best predictors of originality; rather, we hope to provide a new perspective and new insights for future research. The approach can be easily applied in assessing other aspects of writing, which can help raters quantify their subjective experience while scoring originality in essays.

In addition, it must be admitted that it is overly pragmatic to use a three-point scale for ordinal variables Bürkner and Vuorre (2019) and use the statistics, such as average and ICC, which are suitable for interval-scaled variables. The main reason is that when the rating is more than three points, it will be very difficult for human raters to assign a score for originality. Therefore, we followed the operation in Zedelius et al. (2019), using a three-point scale and some descriptive statistical indicators to measure the quality of human scoring. We expect to optimize the objectivity of the originality scoring rubric through the research of computational linguistics, in order to refine more specific descriptions and requirements of rating.

## 5. Conclusions

Our results proved that computational linguistic procedures, to some extent, can predict originality. Topic analysis framed a quantifying context in which the essay to be evaluated and the network-based features offered objective evidence to measure “differences” among essays to be evaluated. Moreover, the results provided a “topic analysis-comparing difference-scoring prediction” channel for originality assessing and showed that a significant performance of features for originality prediction varies across different topics. Furthermore, from a theoretical perspective, the network representation of writing increases our understanding of the relationship between the global connections of words in an essay and writing originality. More useful information and objective evidence can be used to quantify the human rater’s subjective experience and perception in the process of originality scoring.

## Figures and Tables

**Table 1:** Sample size of each prompt.

Prompt	Writing Task	Sample Size		
		Male	Female	Total
Prompt 1	Please write an essay with more than 400 Chinese characters under the title “Company is the best gift”. There is no limit regarding the genre.	98	99	197
Prompt 2	Please write an essay with more than 400 Chinese characters under the title “If we do it again.” There is no limit regarding the genre.	94	97	191
Prompt 3	Please complete the blank in “I forget _____ ” and write an essay with more than 400 Chinese characters under this title. There is no limit regarding the genre.	93	95	188

**Table 2:** Substantive labels are assigned to the final topic solution.

Topic Descriptions		Proportion
Prompt 1		
Topic 1	Some items (such as toys or books) that the writer grew up with	21.16
Topic 2	Family (parents, siblings, or grandparents) that the writer grew up with	21.69
Topic 3	Care and company of friends	21.16
Topic 4	Teachers and students in the class who encouraged and accompanied the writer	17.46
Topic 5	Lack of company, parents were absent for a long time, and the writer hoped to get their attention	18.52
	Prompt 2	
Topic 1	Given another chance, the writer would not give up	18.95
Topic 2	Some things were missed due to fear, which the writer sincerely regrets	3.68
Topic 3	Did some bad things, such as quarreling with family or getting angry	23.16
Topic 4	A commitment to correct mistakes, set goals, and realize dreams (e.g., study hard)	18.95
Topic 5	Reflection, for some reason (for example, being addicted to mobile phones, the writer ignores the people around them)	23.68
Topic 6	Being criticized for making mistakes in school, the writer decided not to do it next time	11.58
Prompt 3		
Topic 1	Forgetting the gratitude and warmth from family made the writer face reality	25.00
Topic 2	Forgetting that persistence and hard work are needed to improve grades and overcome difficulties	13.83
Topic 3	Forgetting an appointment with friends or classmates	18.09
Topic 4	Forgetting the time; forgetting how the writer wanted to get rid of this problem	18.09
Topic 5	Forgetting a large amount of childhood memories that make the writer feel happy	16.49
Topic 6	Forgetting to bring things (e.g., umbrella); forgetting to get the help of classmates or other people	8.51

**Table 3:** R-square for the lasso analysis and the topic quality diagnostic statistics (semantic coherence and exclusivity) cross topics.

Prompt		ALL Topics	Topic 1	Topic 2	Topic 3	Topic 4	Topic 5	Topic 6
1	lambda	0.012	0.036	0.059	0.006	0.084	0.024	——
	R2	0.271	0.360	0.249	0.641	0.305	0.506	——
	Sem.c.	−62.390	−50.550	−50.611	−53.023	−59.908	−52.915	——
	Exclu.	8.197	7.959	8.129	8.396	7.877	8.362	——
2	lambda	0.045	0.070	——	0.091	0.126	0.062	0.013
	R2	0.207	0.551	——	0.297	0.376	0.326	0.686
	Sem.c.	−69.916	−68.902	−112.328	−73.988	−59.809	−78.763	−68.902
	Exclu.	8.469	8.069	9.519	8.343	8.044	8.985	8.069
3	lambda	0.038	0.110	0.142	0.069	0.025	0.043	0.019
	R2	0.197	0.345	0.532	0.465	0.471	0.188	0.635
	Sem.c.	−79.753	−80.058	−70.553	−70.288	−68.474	−74.751	−92.549
	Exclu.	8.560	8.072	8.703	8.412	8.640	8.274	9.140

Note: In Prompt 2, there were only seven essays divided within Topic 2 in LDA analysis; therefore, prediction modeling was not conducted since the quantity is significantly small.

## Data Availability

Not applicable.

## Appendix A

Original Scoring Rubric
Original essay: The idea or story is original, i.e., very different from other essays with the same writing task.
Highly original essay: 3 points ▪ It is clearly different from most other essays ▪ The characters or objects described are clearly different from other essays ▪ The development of the story or plot is clearly different from other essays ▪ The thoughts or feelings expressed are clearly different from other essays ▪ The words, sentences, and idioms used are clearly different from other essays
Moderately original essay: 2 points ▪ Similar to some essays; it may be more novel than some essays ▪ The characters or objects described are not very common ▪ The development of the story or plot is somewhat different from most essays ▪ The expression of thoughts and feelings is not very common and contains some differences with most essays ▪ The words, sentences, and idioms used are different from other essays to a certain extent
Low originality essay: 1 point ▪ Similar to many other essays ▪ The characters or objects described are very common ▪ The development of the story or plot is quite ordinary ▪ The expression of thoughts and feelings are very common ▪ Contains platitudes without new ideas
How to score: First, judge whether the essay is in line with any one of the three-point essay descriptions, and if that is the case, then give the essay a grade of three. If the essay does not meet the requirements, then judge whether the essay is in line with any one of the descriptions of the two-point essay; if it is, rate it as two, and essays that do not meet the above requirements are rated as one. Originality is reflected in a variety of aspects, such as: - The characters or objects described are unusual (for example, the novelty of “book” in “company is the best gift” is higher than “parents”) - An unusual story or plot development (for example, “dad left home early and did not accompany grandma”) - The author expresses very unusual thoughts, feelings, or judgments (but appropriate ones) (for example, latch-key children desire a complete family) - The author chooses very rare words, phrases, texts, or rhetorical styles (for example, several instances of parallelism, personification, or ancient prose) Note: - Off-topic essays are rated as 0; - Do not make ideological or moral judgments.
